# Exploring options for reprocessing of N95 Filtering Facepiece Respirators (N95-FFRs) amidst COVID-19 pandemic: A systematic review

**DOI:** 10.1371/journal.pone.0242474

**Published:** 2020-11-20

**Authors:** Diptanu Paul, Ayush Gupta, Anand Kumar Maurya

**Affiliations:** Department of Microbiology, All India Institute of Medical Sciences, Bhopal, Madhya Pradesh, India; VIT University, INDIA

## Abstract

**Background:**

There is global shortage of Personal Protective Equipment due to COVID-19 pandemic. N95 Filtering Facepiece Respirators (N95-FFRs) provide respiratory protection against respiratory pathogens including SARS-CoV-2. There is scant literature on reprocessing methods which can enable reuse of N95-FFRs.

**Aim:**

We conducted this study to evaluate research done, prior to COVID-19 pandemic, on various decontamination methods for reprocessing of N95-FFRs.

**Methods:**

We searched 5 electronic databases (Pubmed, Google Scholar, Crossref, Ovid, ScienceDirect) and 1 Grey literature database (OpenGrey). We included original studies, published prior to year 2020, which had evaluated any decontamination method on FFRs. Studies had evaluated a reprocessing method against parameters namely physical changes, user acceptability, respirator fit, filter efficiency, microbicidal efficacy and presence of chemical residues post-reprocessing.

**Findings and conclusions:**

Overall, we found 7887 records amongst which 17 original research articles were finally included for qualitative analysis. Overall, 21 different types of decontamination or reprocessing methods for N95-FFRs were evaluated. Most commonly evaluated method for reprocessing of FFRs was Ultraviolet (Type-C) irradiation (UVGI) which was evaluated in 13/17 (76%) studies. We found published literature was scant on this topic despite warning signs of pandemic of a respiratory illness over the years. Promising technologies requiring expeditious evaluation are UVGI, Microwave generated steam (MGS) and based on Hydrogen peroxide vapor. Global presence of technologies, which have been given Emergency use authorisation for N95-FFR reprocessing, is extremely limited. Reprocessing of N95-FFRs by MGS should be considered for emergency implementation in resource limited settings to tackle shortage of N95-FFRs.

**Systematic review identifier:**

PROSPERO, PROSPERO ID: CRD42020189684, (https://www.crd.york.ac.uk/prospero/display_record.php?ID=CRD42020189684).

## Introduction

Global pandemic of Corona Virus Disease of 2019 (COVID-19) has led to over 37 million cases and 1 million deaths worldwide and still counting [1]. It is caused by a novel Corona virus (nCoV), a member of family *Coronaviridae*, now renamed as SARS-CoV-2 [2]. Transmission of this virus occurs through direct, contact and airborne routes, latter particularly when aerosol generating procedures (AGPs) are done during patient care [3]. Consequently, healthcare workers (HCWs) require a full set of personal protective equipment (PPE) including gowns, gloves, facemasks, face-shields or goggles and respirators for their protection during patient care, particularly in intensive care unit settings where AGPs are done regularly [4]. This has created an unprecedented demand for PPEs leading to their global shortage forcing administrative authorities to relook the recommendations of PPE usage in a whole new light [5]. Previously, focus of PPE use strategy was not to share them between patients [6] however, due to this unprecedented crisis, it has radically shifted to optimizing the use of PPEs, their extended use and limited reuse [4, 5]. Respiratory protection is one of the fundamental rights of any employee in workplace. In healthcare settings, HCWs need to be protected against bioaerosols at all costs, which at minimum, is offered by use of N95 Filtering Facepiece Respirator (N95-FFR). These FFRs have a class of filters which is not resistant to degradation by oil and is able to remove 95% particles of 0.3 μm in size, at minimum [7]. They are single use devices ought to be discarded after use to avoid self-inoculation & cross-contamination [8].

Shortage of FFRs is not new, pangs of which were first felt during Severe Acute Respiratory Syndrome (SARS) outbreak in 2003 [9]. The possibility was also predicted for an impending Influenza pandemic consequent to which U.S. Strategic National Stockpile had plans for providing 100 million N95-FFRs nationally, but it was deemed insufficient in event of a longer pandemic [9–11]. Hence, in 2006, Institute of Medicine (IOM) constituted a committee to address reusability of facemasks. Reuse of an FFR was defined as repeatedly donning and doffing of respirator by the same wearer, with or without undergoing reprocessing in between, till it is discarded. The committee recommended reuse of respirators in the event of acute shortage provided they are not obviously damaged or soiled [11]. However, committee specified that no method exists currently for reprocessing of N95-FFRs and identified it as a research priority [11]. Consequently, various research groups began their quest to search a reprocessing method which is efficacious against respiratory pathogens, is safe for human use and maintains the integrity of various components of the respirator. Even after a decade of research, prior to COVID-19 pandemic, no method has been recommended for reprocessing of N95-FFRs. Hence, we conducted this systematic review to determine the status of research done, prior to COVID-19 pandemic, to identify technologies which can be utilized for reprocessing of N95-FFRs in present situation and can be explored in near future to tackle the global crisis of respirator shortage.

## Methods

We report this systematic review (PROSPERO ID: CRD42020189684) in accordance with the Preferred Reporting Items for Systematic Reviews and Meta-Analyses (PRISMA) guidelines [12] and checklist is provided in S1 Table.

### Search strategy

We searched five databases–Pubmed, Google Scholar, Crossref, Ovid and ScienceDirect in May 2020. Grey literature was searched using OpenGrey repository. Search strategies employing combinations of various keywords is provided in S2 Table. Searches in Google Scholar and Crossref were done using Publish or Perish 7 software (Harzing, A.W. 2007) to limit article hits and sort relevant ones. Additionally, we manually searched the back references of included studies and relevant review articles on the topic to identify further eligible studies. Articles in languages other than English were considered only when their abstracts were available in English.

### Eligibility criteria

Original research articles in any language, which evaluated a single or multiple decontamination or reprocessing methods on N95-FFRs were eligible for analysis in this study. Exclusion criteria were (i) Abstracts, posters, review articles, book chapters, letters, guidelines, point of views (ii) articles published in year 2020 and (iii) involving reprocessing or decontamination of other types of masks or respirators such as Gauze, Cloth, Spun-lace, Elastomeric and Powered-air-purifying, only.

### Data extraction

After searching all databases, we exported data in Microsoft® Excel and removed duplicates. Two reviewers (DP & AG) screened titles to remove clearly irrelevant studies. All three reviewers (AG, DP, AKM) independently screened the abstracts and full text of remaining articles to determine final eligibility and resolved any discrepancies through discussion and consensus. After included studies were finalized, data on various variables such as reprocessing method exposure variables, number, type and replicates of FFR models, parameters which were evaluated and final results was entered in Microsoft® Excel independently by all three reviewers. Extracted data was checked and analysed by one reviewer (AG) and disagreements were resolved prior to final analysis.

### Quality assessment

To assess methodological quality and risk bias of studies, a self-developed tool was designed on the basis of STROBE statement [13] due to unavailability of a validated quality assessment tool for such studies. Two authors (AKM and DP) independently assessed the methodological quality and risk bias as per tool. The scheme of scoring and grading of studies is given in S3 Table along with the final quality assessment results. Inter-author concordance on grading of studies was evaluated by third author (AG). Final quality assessment results for included studies, as shown in S3 Table, were prepared by resolving inter-author disagreements by discussion and building consensus.

## Results

### Search results

**Fig 1:**
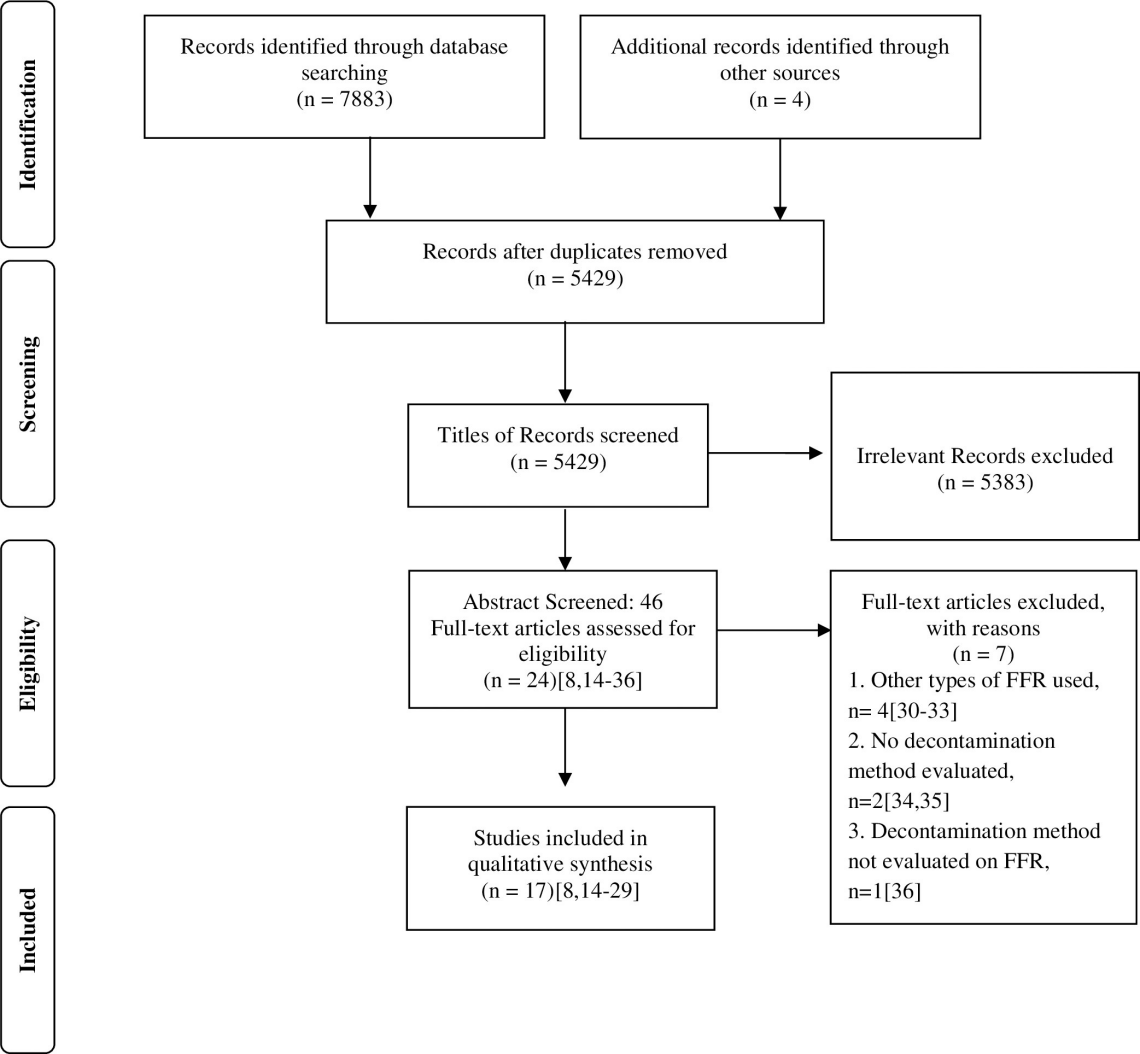
Summary of search, selection and inclusion process. Excluded studies [30–36] Abbreviations: FFR: Filtering Facepiece Respirator, n: Number.

Our search strategy identified 7887 records of which 17 original research articles fit inclusion criteria for qualitative analysis [8, 14–29], methodology of the same has been described in Fig 1. No records were found in OpenGrey database using search strategy.

### Quality assessment

Of 17 studies, 14 were graded as high quality and 3 as moderate quality (S3 Table). Inter-author agreement in grading of studies was 88% (15/17). Overall agreement in quality assessment scores was 64% (11/17).

### Study characteristics

Amongst 17 included studies, 15 were conducted in U.S. [8, 14–27] and 2 in Taiwan [28, 29]. Ten out of 15 studies were conducted by research groups from NIOSH as the principal investigator [8, 14, 16, 17, 21–26], 4 by researchers at Applied Research Associates (ARA) in collaboration with Air Force Research Laboratory at Tyndall Air Force Base, Panama City [18–20, 27] and in 1 study, principal investigators were from University of Nebraska (UoN) [15]. Three studies were an outcome of collaboration between NIOSH, ARA & UoN in various combinations [14, 15, 18]. Two studies from Taiwan were conducted by same researchers at Department of Occupational Safety and Health, Chung Shan Medical University [28, 29]. First study evaluating reprocessing methods for FFRs was published in 2007 [22] and last study in 2018 [29].

### Decontamination/reprocessing methods

**Fig 2:**
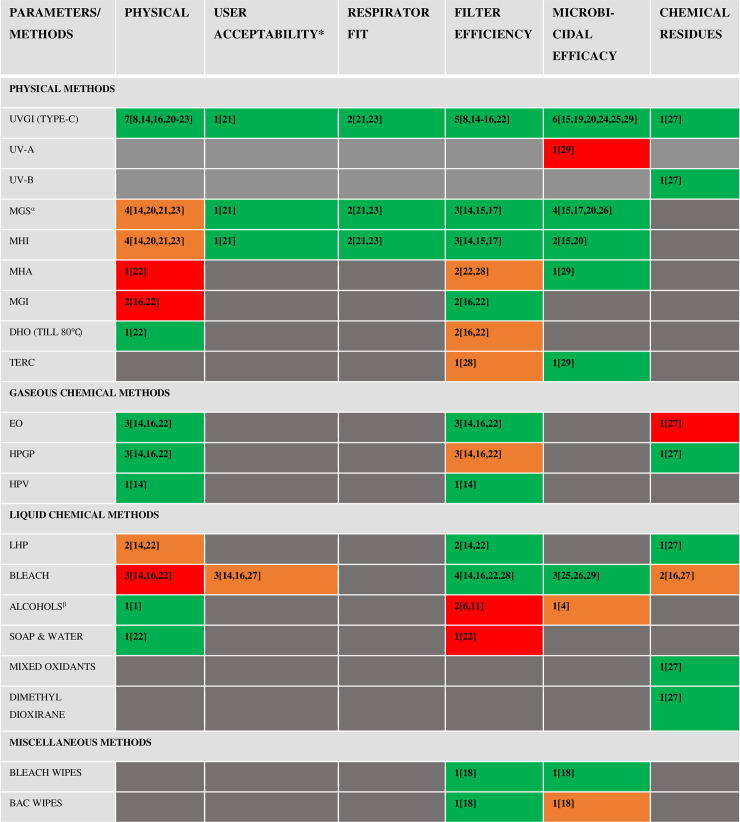
Summary of studies [Total Number, n[Reference] conducted, prior to 2020, on various parameters related to reprocessing of N95 Filtering Facepiece Respirators (FFRs). Coloured cells represent cumulative results of these studies (See Legend Below). Numbers in each coloured cells represent total number of studies conducted on a reprocessing method: parameter combination. Numbers in Parentheses denote the reference number of studies. **Green Cells:** Evidence shows no negative effect of the reprocessing method on the evaluated parameter. **Red Cells:** Evidence shows a negative effect of the reprocessing method on the evaluated parameter. **Orange Cells:** Evidence shows an effect which is either in conflict in different studies or requires careful consideration. **Grey Cells:** No study done on the reprocessing method: parameter combination. * User Acceptability is a composite parameter including odor, wear comfort & donning ease. References 14,16,27 only evaluated odor. α- Fisher *et al* 2011 [17] used Commercial steam bags for generation of steam, other studies used a water reservoir. β- Ethanol (70%) [28, 29] and Isopropyl alcohol (70% [28] and 100% [22]) were used. Abbreviations**: UVGI:** Ultraviolet Irradiation (Type-C, 254 nm), **MGS:** Microwave Generated Steam, **MHI:** Moist heat Incubation in Lab Incubator, **MHA:** Moist Heat in Autoclave, **DHO:** Dry Heat in Oven (Till 80°C), **TERC:** Traditional Electric Rice Cooker, **EO:** Ethylene Oxide, **HPGP:** Hydrogen Peroxide Gas Plasma, **HPV**: Hydrogen Peroxide Vapor, **LHP:** Liquid Hydrogen Peroxide, **BAC:** Benzalkonium Chloride. Note: The summary is only indicative of the collective results of various studies done (prior to 2020) to evaluate effect of reprocessing method on a particular parameter. It doesn’t attempt to endorse or refute any method as the authors strongly believe that there is insufficient data to reach any conclusion.

Overall, 21 different types of decontamination or reprocessing methods for N95-FFRs were evaluated in included studies against various parameters namely physical changes, user acceptability, respirator fit, filter efficiency, microbicidal efficacy and presence of chemical residues post-reprocessing. Number of studies conducted for each reprocessing method, on these parameters are given in Fig 2. Overall, these studies evaluated 9 Physical (Energetic) reprocessing methods namely Ultraviolet (UV-C) Irradiation (UVGI) [8, 14–16, 19, 20, 23–25, 27, 29], UV-A [29], UV-B [27], Moist heat delivered using Microwave generated Steam (MGS) [14, 15, 20, 22, 23, 26], Lab Incubator (MHI) [14, 15, 20, 22, 23] and Autoclave (MHA) [22, 28, 29], Dry heat delivered by Microwave (MGI) [16, 22], Hot Air Oven (DHO) [22] and Traditional Electric Rice Cooker (TERC) [28, 29]; 3 Gaseous chemical decontamination methods namely Hydrogen Peroxide Gas Plasma (HPGP) [14, 16, 22, 27], Hydrogen Peroxide Vapor (HPV) [14] and Ethylene Oxide (EO) [14, 16, 22, 27]; 6 Liquid chemical decontamination methods namely Bleach [14, 16, 22, 25–29], Hydrogen Peroxide (LHP) [14, 22], Alcohols [22, 28, 29], Mixed Oxidants [27], Dimethyl dioxirane [27] and Soap & water [22]; and in one study [18], wipes of Bleach (0.9%), Benzalkonium chloride and Inert substance for surface decontamination of N95-FFRs. Fourteen (14) studies [14–18, 20–29] did comparative evaluation of multiple methods for reprocessing of FFRs whereas in 3 studies only 1 method was evaluated, which was UVGI in all [8, 19, 24]. In 12 studies [14–16, 16–23, 25, 27], intact respirators were exposed to the decontamination method whereas in 5, cut pieces of facepiece portion were exposed [8, 24, 26, 28, 29]. Furthermore, in one study [8], pieces of straps were also exposed separately to UVGI. In 4 studies, FFRs underwent multiple cycles (3 in all studies) of decontamination for reprocessing [14, 17, 18, 23].

### Respirator models

In 10 of 17 studies, the identities of N95-FFR models used was disclosed [8, 15, 17, 19, 21, 23–25, 28, 29], details of which against the reprocessing method and parameters evaluated are given in S4 Table. Overall, 23 different models of N95-FFRs were disclosed in 10 studies, 19 of which are approved as surgical respirators by FDA, whereas 4 are Particulate respirators. All respirators used in these studies, irrespective of whether identities were disclosed or not, were NIOSH approved. 3M1860 [8, 15, 19, 21, 23], 3M1870 [15, 17, 19, 21, 23] & 3M8210 [21, 23, 24, 28, 29] were the most commonly used N95-FFRs, each being used in 5 studies. 3M1860 & 3M1870, both surgical respirators were tested against three reprocessing methods i.e. UVGI, MGS and MHI, where identity was disclosed whereas 3M8210, a particulate respirator was exposed to 7 different reprocessing methods. Furthermore, in 2 studies, P100 respirators were also evaluated but in both identities were not disclosed [16, 22].

### Decontamination methods

#### A. Physical (Energetic) methods

*i*. *Ultra-Violet Irradiation (UVGI)*. Thirteen studies [8, 14–16, 19–25, 27, 29] evaluated exposure to UV-C (254 nm) as a reprocessing method for FFRs, as shown in Fig 2. All 23 known models of N95-FFRs were reprocessed using UV-C in at least one study (S4 Table). Furthermore, one study each also examined the microbiological efficacy of UV-A [29] and presence of chemical residues after using UV-B [27]. Exposure variables of UVGI (UV-C) on N95-FFRs and summary of results are provided in Table 1. Different parameters evaluated against UVGI are detailed in Fig 2. Overall, UVGI has shown to be microbiologically efficacious [15, 19, 20, 24, 25, 29], preserve physical appearance of FFRs [8, 14, 16, 20–23] & their filter efficiency [8, 14–16, 22], acceptable to users in terms of odor, donning ease and wear comfort [21], maintain respirator fit [21, 23] and devoid of any toxic residues post-exposure [27]. UVGI has shown to preserve filter efficiency & achieve adequate microbicidal efficacy post-exposure in 9 [8, 15, 24] & 18 different N95-FFR models [15, 19, 24, 25, 29], respectively, where identity of models was disclosed.

**Table 1:** Summary of characteristics of studies using Ultraviolet Irradiation (UVGI) as a reprocessing method for N95-FFRs.

Authors (Year)		Variables of UVGI Irradiation					Variables of FFRs			Results	
	Type	Irradiance (mW/cm^2^)	Duration	Dose (J/cm^2^)	Sides Exposed to UVGI	No. of Cycle	Total no. of Models used	Part of FFR exposed to UVGI	Repli-cates	Parameters Assessed	Summary of Results
Bergman *et al* [14] (2010)	C	1.8	45 m	-	Outer (Convex)	3	6	Intact	3	Physical Changes	No observable physical changes on FFRs
										Odor	No comment on odor
										Filter Efficiency	Expected levels of Filter Aerosol penetration (<5%) & filter airflow resistance
Lore *et al* [15] (2012)	C	1.6–2.2	15 m	1.8	Outer (Convex)	1	2	Intact	9	Filter Efficiency	No significant degradation of filter performance
										Microbicidal Efficacy	>4 log^10^ TCID_50_/ml reduction of H5N1 Avian Influenza virus
Viscusi *et al* [16] (2009)	C	0.18–0.2	30 m	0.17–0.18	Each side	1	9	Intact	3	Physical Changes	No observable physical changes on FFRs
										Filter Efficiency	Didn’t affect Filter efficiency
Lindsley *et al* [8] (2015)	C			120, 240, 470, 950 (For mask layers);	NA	1	4	Facepiece Coupons and Straps	4	Structural Integrity	Strengths of respirator materials was substantially reduced (in some cases>90%)
										Filter Efficiency	Slight increase in particle penetration but no effect on airflow resistance
				590, 1180, 2360 (For straps, each side)							
Mills *et al* [19] (2018)	C	17	60–70 s	1	Outer (Convex)	1	15	Intact	3	Microbicidal Efficacy	≥3 log_10_ TCID_50_/ml reduction in Influenza virus (H1N1) viability on 12/15 FFR models and straps from 7/15 FFR models
Heimbuch *et al* [20] (2011)	C	1.6–2.2	15 m	1.8	Outer (Convex)	1	6	Intact	3	Physical Changes	No observable physical changes on FFRs
										Microbicidal Efficacy	>4 log_10_ TCID_50_/ml reduction of Influenza virus (H1N1)
Viscusi *et al* [21] (2011)	C	1.8	30 m	-	Each side	3	6	Intact	2	Physical Changes	No observable physical changes on FFR
											No clinically meaningful reduction in respirator fit, increase in odor, increase in discomfort or increased difficulty in donning
										User Acceptability	
										Respirator Fit	
Viscusi *et al* [22] (2007)	C	0.24	15/ 240 m	-	Each side	1	2	Intact	4	Physical Changes	No observable physical changes on FFRs
										Filter Efficiency	Not significantly affected by both time durations on both types of FFRs (N95 and P100)
Bergman *et al* [23] (2011)	C	1.8	15 m	-	Outer (Convex)	3	3	Intact	2	Physical Changes	No observable physical changes on FFRs
										Respirator Fit	No significant changes in Respirator fit
Fisher *et al* [24] (2010)	C	2.5	1, 2, 4, 10 m on 3M 8210,1870	0.03, 0.1 & 0.3 on Wilson, 3M 1860 and KC	Each side	1	6	Facepiece Coupons	3	IFM specific dose for	Log Reduction of MS2 Coliphage is a function of FFR model specific IFM UV-C dose
			10m on Cardinal N95-ML							Microbicidal Efficacy	
Lin *et al* [29] (2018)	C	18.9	1, 2, 5, 10, 20 m	-	NA	1	1	Cut pieces	3	Microbicidal Efficacy	99–100% biocidal efficacy against *Bacillus subtilis* spores
Vo *et al* [25] (2009)	C	0.4	1, 2, 3, 4, 5 hr	1.44, 2.88, 4.32, 5.76, 7.2	One side	1	1	Intact	3	Microbicidal Efficacy	3 log reduction of MS2 Coliphage at dose of 4.32 J/cm^2^ and complete removal at dose of ≥7.2 J/cm^2^
Salter *et al* [27] (2010)	C	3.4	1 hr	27	NA	1	6	Coupons, straps,	3	Presence of Toxic Chemical residues Post-exposure	No toxic residues post-exposure
								Nose cushion,			
								Nose pieces			
Lin *et al* [29] (2018)	A	31.2	1, 2,5, 10, 20 m	-	Each side	1	1	Cut pieces	3	Microbicidal Efficacy	Poor Microbicidal efficacy against *Bacillus subtilis* spores
Salter *et al* [27] (2010)	B	4	1 hr	-	NA	1	6	Coupons, straps, nose cushion,	3	Presence of Toxic Chemical residues Post-exposure	No toxic residues post-exposure
								Nose pieces			

**ABBREVIATIONS: mW/cm**^**2**^: milli Watt per square centimetre, **J/cm**^**2**^: Joules per square centimetre **m:** Minute, **NA:** Not Applicable, **FFR:** Filtering Facepiece Respirator, **TCID:** Tissue Culture Infectious Dose, **s:** Seconds **IFM:** Internal Filtering Media, **hr:** Hour

*ii*. *Moist heat*. Delivering moist heat to FFRs has been evaluated in 10 studies [14, 15, 17, 18, 21–23, 26, 28, 29]. Modalities of exposure involved exposing FFRs to steam created in a microwave (MGS), either by using water reservoir [14, 15, 20, 21, 23, 26] or commercial steam bags [17]; in a lab incubator with a water reservoir heated at 60-70°C (MHI) [14, 15, 18, 21, 23] and by autoclaving at 121°C (MHA) [22, 28, 29]. Parameters evaluated for these treatments are given in Fig 2 and the exposure variables and results of individual studies are described in Table 2. Known FFR models which underwent reprocessing by both MGS and MHI were 3M1860, 3M1870, 3M8000 and 3M8210, whereas, for MHA only known FFR model was 3M8210. MHA physically destroyed FFRs thus deemed unsuitable for further evaluation [22]. Both MGS & MHI methods showed acceptable microbiological efficacy [15, 17, 20, 26] and no significant effect on user acceptability [21], respirator fit [21, 23] and filter efficiency [14, 15, 17], till 3 cycles of decontamination.

**Table 2:** Summary of characteristics of studies using physical decontamination methods, other than UVGI, for reprocessing of FFRs.

	Variables of Decontamination Methods				Variables of FFRs			Results	
Authors (Year)	Mode of Delivery	Temperature	Duration	No. of Deconta-mination Cycle	Total no. of Models used	Part of FFR exposed	Replicates	Parameters Assessed	Summary of Results
**DRY HEAT**									
Viscusi *et al* [16] (2009)	Microwave	-	2 m	1	9	Intact	3	Physical Changes	Observable physical changes on many models of FFRs
			(1 m each side)		(6 N95				
								Filter Efficiency	Expected levels of Filter Aerosol penetration (<5%) & filter airflow resistance
					3 P100)				
Viscusi *et al* [22] (2007)	Microwave	-	2 and 4 m	1	2	Intact	4	Physical Changes	No visible changes after 2 min for both models
					(1 N95				
			(1 & 2 m each side)						
					1 P100)				
									Visible damage after 4 min for both models
								Filter Efficiency	Filter efficiency not significantly changed after 2 min for both models
									Filter efficiency of N95-FFR was significantly increased after 4 min
Viscusi *et al* [16] (2009)	Hot air Oven	80-120° C	1 hr	1	9	Intact	3	Physical Changes	No Comment
					(6 N95 3 P100)				
								Filter Efficiency	Temperature affected filter aerosol penetration and component melting which was model specific
Viscusi *et al* [22] (2007)	Hot air oven	80° C & 160° C	1 hr	1	2	Intact	4	Physical Changes	No visible changes for either type of respirator at 80° C
					(1 N95				
					1 P100)				
									Complete destruction of both types of respirators at 160° C
								Filter Efficiency	
									Small increase in average penetration for both types of respirators
Lin *et al* [28] (2017)	Rice Cooker	149-164° C	3 m	1	1	Cut pieces	3	Filter Efficiency	Decontamination reduced the filter quality but less than liquid chemical methods
Lin *et al* [29] (2018)	Rice Cooker	149-164° C	3 m	1	1	Cut pieces of FFR layers	3	Microbicidal Efficacy	99–100% Biocidal efficacy against *Bacillus subtilis* spores
**MOIST HEAT**									
Bergman *et al* [14] (2010)	Microwave (MGS)		2 m	3	6	Intact	3	Physical Changes	Partial separation of inner foam cushion of 1 FFR model
								Odor	No comment on odor
								Filter Efficiency	Expected levels of filter aerosol penetration (<5%) & filter airflow resistance
Lore *et al* [15] (2012)	Microwave (MGS)		2 m	1	2	Intact	9	Filter Efficiency	No significant degradation of filter performance
								Microbicidal Efficacy	>4 log_10_ TCID_50_/ml reduction of H5N1 Avian Influenza virus
Fisher *et al* [17] (2011)	Microwave (MGS)		90 s	3	3	Intact	3	Microbicidal Efficacy	>3 log_10_ reduction in pfu/FFR of MS2 Coliphage
Heimbuch *et al* [20] (2011)	Microwave (MGS)		2 m	1	6	Intact	3	Physical Changes	Slight separation of foam nose cushion in 1 FFR model
								Microbicidal Efficacy	>4 log_10_ TCID^50^/ml reduction of Influenza virus (H1N1)
Viscusi *et al* [21] (2011)	Microwave (MGS)		2 m	1	6	Intact	2	Physical Changes	Slight separation of inner foam nose cushion in 1 FFR model
								User Acceptability	No significant changes in odor, increase in discomfort or increased difficulty in donning
									Strap breakage during multiple donning not more frequent than in controls
								Respirator Fit	No clinically meaningful reduction in respirator fit
Bergman *et al* [23] (2011)	Microwave (MGS)		2 m	3	3	Intact	2	Physical Changes	Slight separation of inner foam nose cushion in 1 FFR model
								Respirator Fit	No significant changes in Respirator fit
Fisher *et al* [26] (2009)	Microwave (MGS)		15, 30, 45, 60, 75, 90 s	1	1	Cut pieces	4	Microbicidal Efficacy	>4 log_10_ reduction in MS2 Coliphage pfu/ml after ≥ 45 seconds
Bergman *et al* [14] (2010)	Lab Incubator (MHI)	60°C	30 m	3	6	Intact	3	Physical Changes	Partial separation of inner foam cushion of 1 FFR model
								Odor	No comment on odor
								Filter Efficiency	Expected levels of Filter Aerosol penetration (<5%) & filter airflow resistance
Lore *et al* [15] (2012)	Lab Incubator (MHI)	65 ± 5°C	3 hr	1	2	Intact	9	Filter Efficiency	No profound reduction in filter efficiency
								Microbicidal Efficacy	>4 log_10_ TCID_50_/ml reduction of H5N1 Avian Influenza virus achieved
Heimbuch *et al* [20] (2011)	Lab Incubator (MHI)	65 ± 5°C	30 m	1	6	Intact	3	Physical Changes	No obvious signs of deformation or deterioration of FFRs
								Microbicidal Efficacy	>4 log_10_ TCID_50_/ml reduction of Influenza virus (H1N1)
Viscusi *et al* [21] (2011)	Lab Incubator (MHI)	60°C	30 m	1	6	Intact	2	Physical Changes	Slight separation of inner foam nose cushion in 1 FFR model
								User Acceptability	Mean Odor scores were increased only for 1 FFR model
									No significant increase in discomfort or increased difficulty in donning
									Strap breakage during multiple donning not more frequent than in controls
								Respirator Fit	No clinically meaningful reduction in respirator fit
Bergman *et al* [23] (2011)	Lab Incubator (MHI)	60°C	15 m	3	3	Intact	2	Physical Changes	Slight separation of inner foam nose cushion in 1 FFR model
								Respirator Fit	No significant changes in Respirator fit
Viscusi *et al* [22] (2007)	Autoclave (MHA)	121°C	15/ 30 m	1	2	Intact	4	Physical Changes	N95-FFRs were deformed in both conditions and P100 FFRs were unchanged but respirator media felt softer
								Filter Efficiency	Degradation in filter efficiency of both Respirator types
Lin *et al* [28] (2017)	Autoclave (MHA)	121°C	15 m	1	1	Cut pieces of FFR facepiece	3	Filter Efficiency	Decontamination reduced the filter quality but less than liquid chemical methods
Lin *et al* [29] (2018)	Autoclave (MHA)	149-164° C	3 m	1	1	Cut pieces of FFR facepiece	3	Microbicidal Efficacy	99–100% Biocidal efficacy against *Bacillus subtilis* spores

**ABBREVIATIONS: UVGI:** Ultraviolet Irradiation, **FFR:** Filtering Facepiece Respirator, **m:** minute, **hr:** hour, **TCID:** Tissue Culture Infectious Dose, **s:** second**, pfu:** Plaque Forming Unit

*iii*. *Dry heat*. Dry heat for reprocessing of FFRs has been evaluated in 4 studies [16, 22, 28, 29] wherein microwave (MGI) [16, 22], Hot Air Oven (DHO) [16, 22] and Electric Rice Cooker (TERC) [28, 29] have been used. 3M8210 was the only known N95-FFR model which underwent reprocessing by any dry heat delivering method [28, 29]. Various parameters which have been evaluated against them are shown in Fig 2 and their exposure variables and results are summarized in Table 2. In MGI method, respirator models were destroyed in both studies [16, 22]. FFRs reprocessed by DHO were able to physically withstand temperatures at 80°C without affecting durability and filter efficiency [16, 22]. Electric rice cooker (TERC) was able to provide 99–100% biocidal efficacy against *Bacillus subtilis* spores [29].

#### B. Gaseous chemical methods

Only 4 studies [14, 16, 22, 27], prior to 2020, had evaluated a gaseous disinfection method for reprocessing of N95-FFRs. The methods used were Ethylene Oxide (EO) [14, 16, 22, 27], Hydrogen peroxide in a Plasma Sterilizer (HPGP) [14, 16, 22, 27] and Hydrogen Peroxide in vaporized form by using a commercial automated vapor generator [22]. FFR models were not disclosed in any of the studies. Parameters against which they were evaluated; and their exposure variables and findings of the studies are provided in Fig 2 and Table 3, respectively. After EO sterilization, FFRs didn’t showed any physical changes [14, 16, 22], or had offensive odor [14, 16], and filter efficiency was also not degraded significantly [14, 16, 22] even after undergoing 3 cycles [14]. In 3 studies, where HPGP was evaluated, no significant physical changes on the FFRs were noted [14, 16, 22] but filter efficiency of 25% (9/36) respirators was noted to be degraded in one [14] of three [14, 16, 22] studies. However, similar effect was not noted when FFRs were treated with vaporized form [14, 22].

**Table 3:** Summary of characteristics of studies using gaseous chemical methods for reprocessing of FFRs.

Authors	Variables of Decontamination Methods				Variables of FFRs			Results	
	Disinfectant Sterilizer	Packaging Conditions	Duration	No. of Decontamination Cycles	Total no. of Models used	Part of FFR exposed	Replicates	Parameters Assessed	Summary of Results
Bergman *et al* [14] (2010)	Ethylene Oxide	Kept in Tyvek® pouches	1 hr exposure	3	6	Intact	3	Physical Changes	Partial separation of inner foam cushion of 1 FFR model
	Amsco® Eagle® 3017								
			12 hr aeration						
								Odor	No comment on odor
		6 FFR per pouch							
								Filter Efficiency	Expected levels of filter aerosol penetration (<5%) & filter airflow resistance
Viscusi *et al* [16] (2009)	Ethylene Oxide	Individual poly/paper pouch	1 hr exposure	1	9	Intact	3	Physical Changes	No observable physical changes on FFRs
	3 M Steri-Vac 5XL				(6 N95				
			4 hr aeration						
					3 P100)				
								Filter Efficiency	Expected levels of filter aerosol penetration (<5%) & filter airflow resistance
Viscusi *et al* [22] (2007)	Ethylene Oxide	Individual poly/paper pouch	1 hr exposure	1	2	Intact	4	Physical Changes	Straps of P100 FFRs were slightly darkened
	3 M Steri-Vac 4XL & 5 XL								
								Filter Efficiency	Average penetration increased for both respirator types but were within NIOSH certification criteria
			4 hr aeration						
Salter *et al* [27] (2010)	Ethylene Oxide	Individual sterilization pouch	3 hr exposure	1	6	Intact	3	Presence of Toxic Chemical Residues	EO was not detected on any of the model
	Amsco® Eagle® 3017		12 hr aeration						
									Treated EO contained Diacetone alcohol and a possible mutagen and carcinogen, 2-hydroxyethyl acetate (HEA)
Bergman *et al* [14] (2010)	H_2_O_2_ Gas Plasma (HPGP)	Mylar/Tyvek^®^ pouch	55 m cycle time	3	6	Intact	3	Physical Changes	No physical changes on FFRs
								Odor	No comment on odor
	STERRAD® 100S	6 samples per pouch						Filter Efficiency	25% (9/36) samples had aerosol penetration >5% suggestive of degradation in filter efficiency
Viscusi *et al* [16] (2009)	H_2_O_2_ Gas Plasma (HPGP)	Mylar/Tyvek® pouch	55 m cycle time	1	9	Intact	3	Physical Changes	Metallic nose bands not as shiny as unexposed controls
					(6 N95			Filter Efficiency	Expected levels of Filter Aerosol penetration (<5%) & filter airflow resistance
	STERRAD® 100S	6 samples per pouch			3 P100)				
Viscusi *et al* [22] (2007)	H_2_O_2_ Gas Plasma (HPGP)	Mylar/Tyvek® pouch		1	2	Intact	4	Physical Changes	Aluminium nosebands slightly tarnished with both cycles
								Filter Efficiency	Average penetration not significantly increased & remained within limit of NIOSH certification criteria for both respirator types and cycling conditions
	STERRAD® 100S		55 m						
	STERRAD® NX		100 m						
Salter *et al* [27] (2010)	H_2_O_2_ Gas Plasma (HPGP)	Sterilization pouches	55 m	1	6	Intact	3	Presence of Toxic Chemical Residues	No residues on FFRs
									Sterilization cycle aborted when >6 FFRs were loaded in the sterilization chamber
	STERRAD® 100S								
Bergman *et al* [14] (2010)	H_2_O_2_ Vapor (HPV)		15 m dwell	3	6	Intact	3	Physical Changes	No physical changes on FFRs
								Odor	No comment on odor
			125 m total cycle time					Filter Efficiency	Expected levels of filter aerosol penetration (<5%) & filter airflow resistance
	Clarus® R HPV Generator								

**ABBREVIATIONS: FFR:** Filtering Facepiece Respirator, **hr:** Hour, **m:** Minute, **H**_**2**_**O**_**2**_: Hydrogen Peroxide

#### C. Liquid chemical methods

Six different liquid decontamination methods have been evaluated on N95-FFRs in 8 studies [14, 16, 22, 25–29]. These are Bleach [14, 16, 22, 25–29], Liquid Hydrogen Peroxide (LHP) [14, 22, 27], Alcohols [22, 28, 29] including Ethanol and Isopropyl Alcohol, Mixed oxidants [27], Dimethyl Dioxirane [27] and Soap solution [22]. Parameters against which they were evaluated, their exposure variables and results of the studies are provided in Fig 2 and Table 4, respectively. Against Bleach, only known N95-FFR models evaluated were 3M8210 and Wilson SAF-T-FIT Plus (S4 Table). 3M8210 was the only known N95-FFR which was evaluated for Alcohols [28, 29].

**Table 4:** Summary of characteristics of studies using liquid & miscellaneous chemical methods for reprocessing of FFRs.

Authors	Variables of Decontamination Methods				Variables of FFRs			Results	
	Disinfectant	Concentration	Duration	No. of Decontamination Cycles	Total no. of Models used	Part of FFR exposed	Replicates	Parameters Assessed	Summary of Results
Bergman *et al* [14] (2010)	Liquid H_2_O_2_ (LHP)	6%	30 m Submersion	3	6	Intact	3	Physical Changes	Staples were oxidized to varying degree
								Odor	No comment on odor
								Filter Efficiency	Expected levels of Filter Aerosol penetration (<5%) & filter airflow resistance
Viscusi *et al* [22] (2007)	Liquid H_2_O_2_ (LHP)	3%	30 m submersion	1	2	Intact	4	Physical Changes	No observable changes on both respirator types with 3% H_2_O_2_ & slight fading of label ink with 6% H_2_O_2_
					(1 N95				
					1 P100)				
								Filter Efficiency	Average penetration within NIOSH certification limit for both respirator types & both concentrations
		6%							
Salter *et al* [27] (2007)	Liquid H_2_O_2_ (LHP)	3%	30 m submersion	1	6	Intact	3	Presence of Toxic Chemical Residues	No deposition of significant quantities of toxic residues on FFRs
Bergman *et al* [14] (2010)	NaOCl (Bleach)	0.6%	30 m Submersion	3	6	Intact	3	Physical Changes	Metallic nosebands were tarnished, Staples were oxidized to varying degree, discoloured inner nose pads, dry to touch
								Odor	All FFRs had a characteristic bleach odor after overnight air drying
								Filter Efficiency	Expected levels of filter aerosol penetration (<5%) & filter airflow resistance
Viscusi *et al* [16] (2009)	NaOCl (Bleach)	0.6%	30 m Submersion	1	9	Intact	3	Physical Changes	Metallic nose bands were tarnished
								Odor	All FFRs had a scent of bleach and after rehydration with water, increase in chlorine off-gassing was measured
								Filter Efficiency	Expected levels of filter aerosol penetration (<5%) & filter airflow resistance
Lin *et al* [28] (2017)	NaOCl (Bleach)	0.5%	10 m Submersion	1	1	Cut pieces of facepiece	3	Filter Efficiency	Decontamination reduced the filter quality
Viscusi *et al* [22] (2007)	NaOCl (Bleach)	0.52%	30 m Submersion (both)	1	2	Intact	4	Physical Changes	Aluminium nose bands were tarnished at both concentrations
					(1 N95				
		5.2%			1 P100)				
								Filter Efficiency	At 0.52% & 5.2% conc., average penetration for both respirator types were within NIOSH certification criteria
Lin *et al* [29] (2018)	NaOCl (Bleach)	0.54%	NA Inoculated	1	1	Cut pieces of Face-piece	3	Microbicidal Efficacy	100% Biocidal efficacy against *Bacillus subtilis* spores at the lowest concentration
		2.7%							
		5.4%							
Vo *et al* [25] (2009)	NaOCl (Bleach)	0.005/0.01/0.05/0.1/	10 m Submersion	1	1	Intact	3	Microbicidal Efficacy	≥0.5% bleach causes 4 log_10_ reduction in pfu/ml of MS2 Coliphage
		0.25/0.5/							
		0.75%							
Fisher *et al* [26] (2009)	NaOCl (Bleach)	0.0006%, 0.006%, 0.06%, 0.6%	2 m Submersion	1	1	Cut Coupons of Face-piece	3	Microbicidal Efficacy	0.6% bleach causes 4 log_10_ reduction in pfu/ml of MS2 Coliphage
Salter *et al* [27] (2010)	NaOCl (Bleach)	0.6%	30 m Submersion	1	6	Intact	3	Physical changes	Corrosion of metal parts was noted
								Odor	FFRs retained a bleach odor following an off-gas period of 18 hour
								Presence of Toxic Chemical Residues	Measured amount of residual chlorine was below permissible exposure limit
Viscusi *et al* [22] (2007)	Soap & Water	1g/L	2 m	1	2	Intact	4	Physical Changes	No physical changes observed for both durations
			20 m Submersion (both)		(1 N95				
					1 P100)				
								Filter Efficiency	Average penetration increased for both durations and both respirators
Salter *et al* [27] (2007)	Mixed Oxidants	(10% Oxone, 6% Sodium Chloride, 5% Sodium Bicarbonate)	30 m submersion	1	6	Intact	3	Physical Changes	Oxidised metal parts
								Odor	Left distinct odor on FFRs
								Presence of Toxic Chemical Residues	No comment
Salter *et al* [27] (2007)	Dimethyl Dioxirane	(10% Oxone, 10% Acetone, 5% Sodium Bicarbonate)	30 m submersion	1	6	Intact	3	Physical Changes	Oxidised metal parts
								Odor	White residue accumulated on FFRs
								Presence of Toxic Chemical Residues	Left distinct odor on FFRs
									Retained in quantity by all 6 FFRs
**MISCELLANEOUS METHODS**									
Heimbuch *et al* [18]	NaOCl (Bleach) wipes	0.9%	Surface Cleaning of outer and inner layers	3	3	Intact	3	Microbicidal Efficacy	3–5 log reduction of *S*. *aureus* in the presence of mucin
								Filter Efficiency	Mean particle penetration was <5%
								Mucin removal	No mucin detected, likely due to interference in measurement assay by NaOCl
Heimbuch *et al* [18]	BAC wipes		Surface Cleaning of outer and inner layers	3	3	Intact	3	Microbicidal Efficacy	>4 log reduction of *S*. *aureus* in the presence of mucin in most FFR samples
								Filter Efficiency	Mean particle penetration was <5% but more than Bleach
								Mucin removal	Removal efficiency ranged from 21.47–76.41% but was poorer than inert wipes
Heimbuch *et al* [18]	Inert wipes		Surface Cleaning of outer and inner layers	3	3	Intact	3	Microbicidal Efficacy	No antibacterial activity
								Filter Efficiency	Mean particle penetration was <5%
								Mucin Removal	Removal efficiency ranged from 21.47%-76.41% and better than BAC wipes

**ABBREVIATIONS: FFR:** Filtering Facepiece Respirator, **H**_**2**_**O**_**2**_: Hydrogen Peroxide, **m:** Minute, **NaOCl:** Sodium Hypochlorite, **NIOSH:** National Institute of Occupation Safety & Hygiene, **g/L:** Gram/Liter, ***S*. *aureus*:** *Staphylococcus aureus*, BAC: Benzalkonium Chloride

#### D. Miscellaneous methods

In one study [18], commercial wipes of 0.9% Sodium Hypochlorite, Benzalkonium Chloride and an Inert material were evaluated for changes in filter efficiency and microbicidal efficacy by applying them on surface of N95-FFRs, as shown in Fig 2 & Table 4.

## Discussion

An Influenza pandemic was always on the horizon and in 2009, it became reality. Researchers at NIOSH have been looking actively for finding a suitable method for reprocessing of FFRs since 2006 after the report of IOM Committee to tackle global shortage of FFRs [11, 22]. Consequently, search for a suitable reprocessing method began under NIOSH. During 2007–2012, 12 studies were published which evaluated reprocessing methods for FFRs, most of them were conducted by or in collaboration with NIOSH [14–17, 20–27]. In contrast, between 2013–2019, only 5 published studies had evaluated a reprocessing technique for N95-FFRs [19, 20, 28, 29], with last study published by NIOSH in 2015 [8]. Ongoing COVID-19 pandemic has brutally exposed the stalled progress in research to address this issue.

It has been shown that the surface stability of SARS-CoV-2 on various surfaces lasts up to 3 days but this study didn’t include porous surfaces like that of respirators [37]. However, a study recently, showed it to be present on outer layer of surgical masks on day 7 [38]. This recent data makes it imperative to decontaminate FFRs in between use as the risk of contact transmission without decontamination is considerable. Previously, CDC also discouraged reusing N95-FFRs whenever risk of contact transmission of a pathogen was high [6]. Furthermore, it is in larger global interest to find a suitable reprocessing method for N95-FFRs as they are not used frequently by HCWs in low to middle income countries (LMICs) while tackling airborne pathogens, such as *Mycobacterium tuberculosis*, against which their use is mandatory [39, 40]. Finding a reprocessing method for FFRs will lead to provision of adequate respiratory protection for HCWs in such resource limited settings.

We found that UVGI was the most frequently evaluated reprocessing method for N95-FFRs, as shown in Fig 2 and Table 3. Reprocessing by UVGI method maintained the overall physical structure and filter efficiency of the FFRs and was able to demonstrate sufficient microbicidal efficacy. Furthermore, it had insignificant influence on the respirator fit and reprocessed FFRs were devoid of any toxic residues but studies which evaluated these parameters were few. Furthermore, these findings should be assessed in view of varying exposure variables of UV dose used in these studies and the methodological variations in estimating the measures of microbicidal efficacy, as shown in Table 1.

Dose of irradiation is the most important variable for determining microbiological efficacy of UVGI method which, in turn, is determined by irradiance at the surface of FFR and duration of exposure [19]. All studies [15, 19, 20, 24, 25], except one [29], which evaluated the microbicidal efficacy of UVGI used enveloped viruses as the challenge micro-organism. Total doses around 1–2 J/cm^2^ have shown to provide ≥4 log_10_ reduction of viruses inoculated on FFRs [15, 20, 25]. Lin *et al* [29] used *Bacillus subtilis* spores as challenge micro-organisms and found that from a 18.9mW/cm^2^ UV-C source, exposure for 5 min (corresponding to a dose of 5–6 J/cm^2^) was able to kill all spores. However, this study measured relative survival of spores (in percentage) on exposed respirator coupons as compared to control coupons (unexposed) instead of log reduction of spores. Whether such doses will be effective against other airborne pathogens, such as *M*. *tuberculosis* should be assessed in future research. Furthermore, a study by Fisher *et al* [24] concluded that the UV-C dose required for microbicidal efficacy is a function of the dose available to the electret medium rather than total dose, which in turn, is dependent on the penetrance (transmittance) of the layer above it. Hence, effective doses of UV-C for microbicidal efficacy will be model specific and needs to be established accordingly. We conclude that UVGI has great potential to be utilized as an effective decontamination method for N95-FFRs during this time of crisis however, more studies are needed to validate the various variables associated with the delivery of the UVGI method and respirator model specific doses will need to be established.

MGS & MHI methods delivered moist heat to FFRs in a microwave and a bench top laboratory incubator, respectively and have shown no significant effect on user acceptability, respirator fit and filter efficiency till 3 cycles of decontamination [14, 15, 21, 23]. However, multiple studies evaluating physical changes noticed partial separation of inner foam nose cushion in both methods for a particular FFR model (3M1870), where model identity was disclosed, but effect was not pronounced after undergoing multiple cycles of decontamination [14, 23]. Whether it is a model specific issue or not should be evaluated in future studies. In terms of microbicidal efficacy, ≥4 log_10_ reduction of enveloped viruses was demonstrated for both methods [15, 17, 20, 26]. We are of opinion that these methods are low cost, easily doable in any setting, but require more validation in terms of other respirator models and cycles of decontamination, in future studies. MGS method is particularly suitable for implementation by individuals at home and smaller healthcare settings. Sparking due to placing metallic components in microwave has been a concern but it has not been noticed in MGS method [14].

Few studies were done on Dry heat as a modality to reprocess FFRs [16, 22, 28, 29]. Physical degradation of the respirators was noted, in varying degree, with these methods using Microwave (MGI), Hot air oven (DHO) and traditional electric rice cooker (TERC). Of these, TERC has shown to be microbiologically efficacious against *B*. *subtilis* spores and preserve physical architecture and filter efficiency of the respirators in limited studies conducted using it [28, 29] We opine that the literature is insufficient to either recommend or refute dry heat as a method of reprocessing for FFRs.

Ethylene oxide (EO) and Hydrogen peroxide (H_2_O_2_) are ideally suited for reprocessing of temperature sensitive articles hence, their use for reprocessing N95-FFRs is particularly promising. They have been evaluated as a reprocessing method for N95-FFRs simultaneously in 4 studies [14, 16, 22, 27] in which, FFRs were exposed to EO and H_2_O_2_ (HPGP) in their respective sterilizers for standard cycling conditions, as described in Table 3. In addition, Viscusi *et al* [22] evaluated vaporized H_2_O_2_ (HPV) generated in a commercial, automated vapor generator (BIOQUELL®). FFR models were not disclosed in any of these studies. The studies found that EO performed suitably in maintaining the physical architecture and filtration efficiency of the respirators however microbicidal efficacy, user acceptability and effect of respirator fit on N-95 FFRs were not evaluated in any study. Furthermore, a study by Salter *et al* [27] found possible carcinogen and mutagen, 2-hydroxyethyl acetate (HEA) on FFRs which had undergone EO sterilization. Hence, this method cannot be recommended for reprocessing of N95-FFRs due to safety concerns and improving the safety profile of EO by increasing aeration duration post-sterilization can be explored in future studies.

Hydrogen peroxide provides microbicidal activity by way of generating free radicals and its degradation products are safe. In 3 studies, where HPGP was evaluated, no significant physical changes on the FFRs were noted [14, 16, 22] but one study [14] noted degradation in filter efficiency of 25% (9/36) respirators. However, this effect was not noted when FFRs were treated with vaporized form [22, 41]. In a commercial evaluation done for FDA by Batelle Institute on Clarus C HPV generator (BIOQUELL®) in 2016, no filter degradation was noted on 3M1870 FFR even after undergoing 50 cycles of decontamination [41]. This system has been granted emergency use authorization (EUA) by FDA, after COVID-19 pandemic, for reprocessing N95-FFRs [42]. Concerns have been raised regarding throughput of HPGP as in a study authors noticed cycles were aborted in STERRAD® Sterilizer whenever >6 FFRs were placed [27]. This could be due to presence of cellulose in the straps of the respirators leading to absorption of H_2_O_2_ [27]. Prior to 2020, no study, in published literature, had evaluated microbicidal efficacy of H_2_O_2_ on FFRs, but recently, Fisher *et al* [43] found it effective in removing SARS-CoV-2 from N95-FFRs. Furthermore, Batelle report, also showed 6 log reduction of *Geobacillus stearothermophilus* spores on FFRs which underwent reprocessing by HPV [41]. Overall, Hydrogen peroxide in gaseous form is a suitable option for reprocessing N95-FFRs but it needs to be evaluated rigorously for other parameters such as respirator fit and also against other N95-FFR models. However, at present its availability is restricted to limited resource rich settings.

Submersion of FFRs in liquid disinfectants is a simple method of decontaminating them. Bleach was the most frequently evaluated liquid disinfectant for reprocessing of FFRs, being evaluated in 9 studies [14, 16, 18, 22, 25–29] of which, 1 used disinfectant wipes [18]. Exposure to bleach caused physical changes in the FFRs in terms of being stiff, mottled and tarnishing of metallic nosepiece [14, 16, 18, 22]. Offensive odor from FFRs was noticed in most studies [14, 16, 27]. Furthermore, chlorine release has been noted when respirators were exposed to moisture, raising concerns regarding the safety of this method if a person breathes through it [16, 27]. Though it has been found to have no significant degradation in the filter quality of the FFRs [14, 16, 18, 22] and have excellent microbicidal efficacy [18, 25, 26, 29], FFRs decontaminated by bleach are not safe.

Liquid Hydrogen peroxide (LHP) in 3% concentration was able to preserve filter efficiency & physical architecture [14, 22] of the N95-FFRs and was devoid of any toxic residues post-exposure [27]. Alcohols (Ethanol and Isopropyl alcohol) have also been evaluated in 3 studies, but they are known to significantly degrade the filter efficiency due to removal of electrostatic charges from the electret media [22, 28, 29]. Similarly, soap & water degraded the filter efficiency, as noted in a study [22].

We found that UVGI was the most widely evaluated reprocessing method, being evaluated for 23 different known FFR models. Nine known FFR models preserved their filter efficiency and 18 known FFR models achieved adequate microbicidal efficacy after undergoing reprocessing by UVGI method. However, the same FFR model: Parameter combination for UVGI was not evaluated in more than two studies. Six known FFR models were reprocessed by MGS [17, 21, 23] & MHI [21, 23] methods. Except for 3M1870, as discussed previously, none of the FFRs showed physical changes after undergoing reprocessing. In none of the studies which evaluated Gaseous chemical methods, identity of FFR models was disclosed. Thus, we suggest that future studies should include multiple known FFR models while evaluating a reprocessing method as compatibility of the FFR with the reprocessing method is of paramount importance.

A summary assessment of the body of literature, published prior to 2020, on reprocessing of N95-FFRs has been provided in Fig 2. However, the findings of this systematic review and opinion of the authors should be assessed in light of limited literature available on this topic, prior to 2020. Furthermore, readers should also consider the variability in exposure variables of the reprocessing methods and methodological variabilities in the evaluated parameters within and between reprocessing methods. For example, to evaluate microbicidal efficacy, studies have used different categories of micro-organisms and growth parameters accordingly while few included additional soiling challenges to mimic micro-organisms in human secretions. Some parameters were evaluated only in few studies such as odor, wear comfort, and donning ease were evaluated objectively only in 1 study [21], respirator fit in 2 studies [21, 23] and chemical safety in 1 study [27]. Hence, changes in these parameters which are not studied much, nevertheless are important, should be the focus of future studies. We didn’t do a meta-analysis as the number of studies done to evaluate a particular parameter for a reprocessing method were few and heterogeneous in terms of both exposure & methodological variables.

As we write this review, a large body of literature on reprocessing of N95-FFRs has been already published [43–57], but when we did literature search, only few studies were published [44, 45, 49, 57] and majority were in preprint, non-peer reviewed versions. Hence, in this systematic review, we only included studies which were published prior to COVID pandemic. This review may help administrators, infectious disease specialists and infection control personnel to formulate policies for effective utilization of single use, N95-FFRs to prevent respiratory transmission of SARS-CoV-2 as well as other airborne pathogens. It will help researchers to find existing knowledge gaps in respirator reprocessing techniques and help them to design future studies. Furthermore, manufacturers may find it useful by knowing existing limitations and work their way around by developing new respirator material or design, more amenable to commonly available reprocessing techniques.

## Conclusions

We found that published literature on evaluation of reprocessing methods of FFRs was scant, prior to COVID pandemic. Physical methods of decontamination, such as using heat or radiation, were the most commonly evaluated methods for reprocessing of FFRs. Majority of studies evaluated either physical changes or effect on filter efficiency of respirators after undergoing decontamination and the microbicidal efficacy of the decontamination method. Only few studies evaluated the effect of decontamination methods on respirator fit or their chemical safety profile. We found that there was a lot of heterogeneity amongst the studies regarding the exposure variables of UVGI method, used respirator models and methodology to evaluate microbicidal efficacy in terms of challenge micro-organisms, method of exposure of challenge micro-organism to FFRs, use of a soiling challenge and evaluated parameters.

We found that UVGI was the most commonly evaluated method in the published literature, prior to 2020 and it ticks all the boxes required for an ideal reprocessing method for N-95 FFRs. However, doses of UV-C irradiation which can achieve satisfactory microbicidal efficacy needs to be determined specifically for each FFR model. Majority of heat-based methods caused physical changes in the respirators, in varying degree, but adequately removed viral micro-organisms from the surface of FFRs without compromising filter efficiency, even after undergoing multiple cycles of decontamination. In particular, MGS method had extremely short cycle time & seems easy to implement in any setting. Few studies evaluated gaseous chemical methods such as EO and Hydrogen peroxide & found that filter efficiency of FFRs was maintained. However, safety concerns were raised on reusing FFRs which underwent reprocessing by EO, in the only study evaluating it.

To summarize, reusing N95-FFRs is need of the hour due to COVID-19 pandemic. Choosing a reprocessing method for FFR decontamination requires careful considerations of various factors such as physical changes, respirator fit, filter efficiency and chemical safety profile, besides being microbiologically efficacious. Furthermore, compatibility of reprocessing method with the FFR models used in a setting, duration of reprocessing cycle and costs involved make it an extremely complex decision for the infection control personnel and administrators. Presently, promising technologies which need to be evaluated rigorously include UVGI, HP, MGS & MHI. Though, emergency use approvals have been given to Hydrogen Peroxide STERRAD® Gas Plasma Sterilizer and BIOQUELL® Clarus C HPV generator, their presence is extremely limited worldwide, particularly in LMICs. Finding a suitable reprocessing method for N95-FFRs is also important from the perspective of infection control against airborne pathogens in LMICs, such as *Mycobacterium tuberculosis*. MGS and MHI have shown to be efficacious against enveloped viruses and not compromise the filter efficiency up to 3 cycles of decontamination, in multiple studies. Of them, MGS has an extremely short cycle and should be considered for emergency implementation in resource limited settings.

## Supporting information

S1 Table PRISMA checklist. (DOCX)

S2 Table Search strategy. (DOCX)

S3 Table Results of quality assessment & risk bias of included studies (after inter-author agreement). (DOCX)

S4 Table Summary of various reprocessing parameters evaluated for specific FFR models (where disclosed in included studies) by various reprocessing methods. (DOCX)

## References

[1] WHO. COVID-19 situation reports. https://www.who.int/emergencies/diseases/novel-coronavirus-2019/situation-reports. (accessed Oct 12, 2020).

[2] WHO. Naming the coronavirus disease (COVID-19) and the virus that causes it. https://www.who.int/emergencies/diseases/novel-coronavirus-2019/technical-guidance/naming-the-coronavirus-disease-(covid-2019)-and-the-virus-that-causes-it (accessed Jun 27, 2020).

[3] WHO. Modes of transmission of virus causing COVID-19: implications for IPC precaution recommendations. https://www.who.int/news-room/commentaries/detail/modes-of-transmission-of-virus-causing-covid-19-implications-for-ipc-precaution-recommendations (accessed Jun 27, 2020).

[4] WHO. Rational use of personal protective equipment for coronavirus disease (‎‎‎‎‎‎COVID-19)‎ ‎‎‎‎‎ and considerations during severe shortages: interim guidance, 6 April 2020. https://apps.who.int/iris/handle/10665/331695 (accessed Jun 27, 2020).

[5] CDC. Recommended Guidance for Extended Use and Limited Reuse of N95 Filtering Facepiece Respirators in Healthcare Settings—NIOSH Workplace Safety and Health Topic. 2020; Published May 15. https://www.cdc.gov/niosh/topics/hcwcontrols/recommendedguidanceextuse.html (accessed June 29, 2020).

[6] CDC. 2007 Guideline for Isolation Precautions: Preventing Transmission of Infectious Agents in Healthcare Settings. Update: July 2019. https://www.cdc.gov/infectioncontrol/pdf/guidelines/isolation-guidelines-H.pdf (accessed Jun 28, 2020).

[7] CDC. The National Institute for Occupational Safety and Health (NIOSH)- NIOSH guide to the selection and use of particulate respirators. 1996; published online Jan 96. https://www.cdc.gov/niosh/docs/96-101/default.html (accessed Oct 3, 2020).

[8] Lindsley W, Martin S Jr, Thewlis R, Sarkisin K, Nwoko JO, Mead KR, et al Effects of ultraviolet germicidal irradiation (UVGI) on N95 respirator filtration performance and structural integrity. *J Occup Environ Hyg* 2015;12:509–517. 10.1080/15459624.2015.1018518 25806411 PMC4699414

[9] Rubinson L, Nuzzo JB, Talmor DS, O'Toole T, Kramer BR, Inglesby TV. Augmentation of hospital critical care capacity after bioterrorist attacks or epidemics: Recommendations of the Working Group on Emergency Mass Critical Care. *Crit Care Med* 2005;9:311–3. 10.1097/01.ccm.0000173411.06574.d5 16215397

[10] Roberge RJ. Effect of surgical masks worn concurrently over N95 filtering facepiece respirators: extended service life versus increased user burden. *J Public Health Manag Pract* 2008;14:e19–26. 10.1097/01.PHH.0000311904.41691.fd 18287908

[11] Reusability of Facemasks During an Influenza Pandemic: Facing the Flu. Washington, D.C: The National Academies Press; 2006.

[12] Moher D, Liberati A, Tetzlaff J, Altman DG, Group TP. Preferred Reporting Items for Systematic Reviews and Meta-Analyses: The PRISMA Statement. *PLoS Med* 2009;6: e1000097 10.1371/journal.pmed.1000097 19621072 PMC2707599

[13] STROBE Statement: Home. https://www.strobe-statement.org/index.php?id=strobe-home (accessed Jun 18, 2020).

[14] Bergman M, Viscusi D, Heimbuch BK, Wander JD, Sambol Anthony R, Shaffer RE. Evaluation of multiple (3-cycle) decontamination processing for filtering facepiece respirators. *J Eng Fiber Fabr* 2010;5:33–40. 10.1177/155892501000500405

[15] Lore MB, Heimbuch BK, Brown TL, Wander JD, Hinrichs SH. Effectiveness of three decontamination treatments against influenza virus applied to filtering facepiece respirators. *Ann Occup Hyg* 2012;56:92–101. 10.1093/annhyg/mer054 21859950

[16] Viscusi D, Bergman M, Eimer B, Shaffer R. Evaluation of five decontamination methods for filtering facepiece respirators. *Ann Occup Hyg* 2009;53:815–827. 10.1093/annhyg/mep070 19805391 PMC2781738

[17] Fisher E, Williams J, Shaffer R. Evaluation of microwave steam bags for the decontamination of filtering facepiece respirators. *PLoS One* 2011;6:e18585 10.1371/journal.pone.0018585 21525995 PMC3078131

[18] Heimbuch B, Kinney K, Lumley A, Harnish D, Bergman M, Wander J. Cleaning of filtering facepiece respirators contaminated with mucin and Staphylococcus aureus. *Am J Infect Control* 2014;42:265–270. 10.1016/j.ajic.2013.09.014 24462175 PMC4469386

[19] Mills D, Harnish D, Lawrence C, Sandoval M, Heimbuch B. Ultraviolet germicidal irradiation of influenza contaminated N95 filtering facepiece respirators. *Am J Infect Control* 2018;46:e49–e55. 10.1016/j.ajic.2018.02.018 29678452 PMC7115285

[20] Heimbuch B, Wallace W, Kinney K, Lumley AE, Chang YW, Woo MH, et al A pandemic influenza preparedness study: use of energetic methods to decontaminate filtering facepiece respirators contaminated with H1N1 aerosols and droplets. *Am J Infect Control* 2011;39:e1–9. 10.1016/j.ajic.2010.07.004 21145624

[21] Viscusi D, Bergman M, Novak, Faulkner KA, Palmiero A, Powell J, et al Impact of three biological decontamination methods on filtering facepiece respirator fit, odor, comfort, and donning ease. *J Occup Environ Hyg* 2011;8:426–436. 10.1080/15459624.2011.585927 21732856

[22] Viscusi DJ, King WP, Shaffer RE. Effect of Decontamination on the Filtration Efficiency of Two Filtering Facepiece Respirator Models. *J Int Soc Respir Prot* 2007;24:93–107.

[23] Bergman MS, Viscusi DJ, Palmiero AJ, Powell JB, Shaffer RE. Impact of Three Cycles of Decontamination Treatments on Filtering Facepiece Respirator Fit. *J Int Soc Respir Prot* 2011;28:48–57.

[24] Fisher EM, Shaffer RE. A method to determine the available UV-C dose for the decontamination of filtering facepiece respirators. *J Appl Microbiol* 2011;110:28795 10.1111/j.1365-2672.2010.04881.x 21054699 PMC9728109

[25] Vo E, Rengasamy S, Shaffer R. Development of a test system to evaluate procedures for decontamination of respirators containing viral droplets. *Appl Environ Microbiol* 2009;75:7303–9. 10.1128/AEM.00799-09 19801477 PMC2786399

[26] Fisher E, Rengasamy S, Viscusi D, Vo E, Shaffer R. Development of a test system to apply virus-containing particles to filtering facepiece respirators for the evaluation of decontamination procedures. *Appl Environ Microbiol* 2009;75:1500–7. 10.1128/AEM.01653-08 19139225 PMC2655466

[27] Salter WB, Kinney K, Wallace WH, Lumley AE, Heimbuch BK, Wander JD. Analysis of residual chemicals on filtering facepiece respirators after decontamination. *J Occup Environ Hyg* 2010;7:437–45. 10.1080/15459624.2010.484794 20526947 PMC7196687

[28] Lin T, Chen C, Huang S, Kuo C, Lai C, Lin W. Filter quality of electret masks in filtering 14.6–594 nm aerosol particles: Effects of five decontamination methods. *PloS One* 2017;12:e0186217 10.1371/journal.pone.0186217 29023492 PMC5638397

[29] Lin T, Tang F, Hung P, Hua Z, Lai C. Relative survival of Bacillus subtilis spores loaded on filtering facepiece respirators after five decontamination methods. *Indoor Air* 2018; 28:754–762. 10.1111/ina.12475 29855107 PMC7165566

[30] MacIntyre CR, Seale H, Dung TC, et al A cluster randomised trial of cloth masks compared with medical masks in healthcare workers. *BMJ Open* 2015; 5:e006577–e006577. 10.1136/bmjopen-2014-006577 25903751 PMC4420971

[31] Wood GO, Snyder JL. Estimating Reusability of Organic Air-Purifying Respirator Cartridges. *J Occup Environ Hyg* 2011;8:609–17. 10.1080/15459624.2011.606536 21936700

[32] Subhash SS, Cavaiuolo M, Radonovich LJ, Eagan Aaron, Lee ML, Campbell S, et al Effectiveness of common healthcare disinfectants against H1N1 influenza virus on reusable elastomeric respirators. *Infect Control Hosp Epidemiol* 2014;35:894–7. 10.1086/676863 24915224

[33] Lawrence C, Harnish DA, Sandoval-Powers M, Mills D, Bergman M, Heimbuch BK. Assessment of half-mask elastomeric respirator and powered air-purifying respirator reprocessing for an influenza pandemic. *Am J Infect Control* 2017; 45:1324–30. 10.1016/j.ajic.2017.06.034 28844381 PMC6193495

[34] Duarte LRP, Miola CE, Cavalcante NJF, Bammann RH. Maintenance status of N95 respirator masks after use in a health care setting. *Rev Esc Enferm USP* 2010;44:1011–6. 10.1590/s0080-62342010000400022 21337784

[35] Fisher EM, Richardson AW, Harpest SD, Hofacre KC, Shaffer RE. Reaerosolization of MS2 bacteriophage from an N95 filtering facepiece respirator by simulated coughing. *Ann Occup Hyg* 2012;56:315–25. 10.1093/annhyg/mer101 22127875 PMC7537697

[36] Vuma CD, Manganyi J, Wilson K, Rees D. The effect on fit of multiple consecutive donning and doffing of N95 filtering facepiece respirators. *Ann Work Expo Health* 2019;63:930–6. 10.1093/annweh/wxz060 31504129

[37] van Doremalen N, Bushmaker T, Morris DH, et al Aerosol and surface stability of SARS-CoV-2 as compared with SARS-CoV-1. *N Engl J Med* 2020;382:1564–7. 10.1056/NEJMc2004973 32182409 PMC7121658

[38] Chin AWH, Chu JTS, Perera MRA, et al Stability of SARS-CoV-2 in different environmental conditions. *Lancet Microbe* 1:e10 10.1016/S2666-5247(20)30003-3 32835322 PMC7214863

[39] Engelbrecht MC, Kigozi G, Rensburg APJ van, Rensburg DHCJ van. Tuberculosis infection control practices in a high-burden metro in South Africa: A perpetual bane for efficient primary health care service delivery. *Afr J Prim Health Care Fam Med* 2018;10:e1–6. 10.4102/phcfm.v10i1.1628 PMC6018120 29943601

[40] Woith W, Volchenkov G, Larson J. Barriers and motivators affecting tuberculosis infection control practices of Russian health care workers. *Int J Tuberc Lung Dis* 2012;16:1092–6. 10.5588/ijtld.10.0779 22687261 PMC3685429

[41] Battelle. Final Report for the Bioquell Hydrogen Peroxide Vapor (HPV) decontamination for reuse of N95 respirators. 2016; Available from: https://www.fda.gov/emergency-preparedness-and-response/mcm-regulatory-science/investigating-decontamination-and-reuse-respirators-public-health-emergencies (accessed June 28, 2020).

[42] Battelle CCDS Critical Care Decontamination SystemTM being deployed to meet urgent need for personal protective equipment for Nation’s healthcare workforce. Battelle. https://www.battelle.org/newsroom/press-releases/press-releases-detail/battelle-ccds-critical-care-decontamination-system-being-deployed-to-meet-urgent-need-for-personal-protective-equipment-for-nation-s-healthcare-workforce (accessed June 28, 2020).

[43] Fischer RJ, Morris DH, van Doremalen N, et al Effectiveness of N95 respirator decontamination and reuse against SARS-CoV-2 virus. *Emerg Infect Dis* 2020;26:2253–55. 10.3201/eid2609.201524 32491983 PMC7454118

[44] Li DF, Cadnum JL, Redmond SN, Jones LD, Pearlmutter B, Haq MF, et al Steam treatment for rapid decontamination of N95 respirators and medical face masks. *Am J Infect Control* 2020;48:855–857. 10.1016/j.ajic.2020.05.009 32417321 PMC7227495

[45] Schwartz A, Stiegel M, Greeson N, et al Decontamination and reuse of N95 respirators with hydrogen peroxide vapor to address worldwide personal protective equipment shortages during the SARS-CoV-2 (COVID-19) pandemic. *Appl Biosaf* 2020;25:67–70. 10.1177/1535676020919932 PMC9387741 36035079

[46] Kenney P, Chan BK, Kortright K, et al Hydrogen peroxide vapor sterilization of N95 respirators for reuse. *medRxiv* 2020 10.1177/1535676020919932 PMC8185421 33557979

[47] Kumar A, Kasloff SB, Leung A, et al N95 Mask Decontamination using standard hospital sterilization technologies. *medRxiv* 2020 10.1101/2020.04.05.20049346

[48] Zulauf KE, Green AB, Ba ANN, et al Microwave-Generated steam decontamination of N95 respirators utilizing universally accessible materials. *mBio* 2020;11:e00997–20. 10.1128/mBio.00997-20 32587063 PMC7317796

[49] Cadnum JL, Li DF, Redmond SN, John AR, Pearlmutter B, Donskey CJ. Effectiveness of Ultraviolet-C light and a high-level disinfection cabinet for decontamination of N95 respirators. *Pathog Immun* 2020;5:52–67. 10.20411/pai.v5i1.372 32363254 PMC7192214

[50] Grinshpun SA, Yermakov M, Khodoun M. Autoclave sterilization and ethanol treatment of re-used surgical masks and N95 respirators during COVID-19: Impact on their performance and integrity. *J Hosp Infect* 2020; published online Jun 26. 10.1016/j.jhin.2020.06.030 32599011 PMC7320268

[51] Xiang Y, Song Q, Gu W. Decontamination of surgical face masks and N95 respirators by dry heat pasteurization for one hour at 70°C. *Am J Infect Control* 2020; published online May 30. 10.1016/j.ajic.2020.05.026 32479844 PMC7260521

[52] Jatta M, Kiefer C, Patolia H. N95 Reprocessing by low temperature sterilization with 59% vaporized hydrogen peroxide during the 2020 COVID-19 Pandemic. *Am J Infect Control* 2020; published online Jun 26. 10.1016/j.ajic.2020.06.194 32599102 PMC7319649

[53] Li DF, Cadnum JL, Redmond SN, Jones LD, Donskey CJ. It’s not the Heat, it’s the humidity: Effectiveness of a rice cooker-steamer for decontamination of cloth and surgical face masks and N95 Respirators. *Am J Infect Control* 2020;48:854–855. 10.1016/j.ajic.2020.04.012 32334003 PMC7174981

[54] Saini V, Sikri K, Batra SD, Kalra P, Gautam K. Development of a Highly effective low-cost vaporized hydrogen peroxide-based method for disinfection of personal protective equipment for their selective reuse during pandemics. *Gut Pathog* 2020;12: 29 Published online Jun 19. 10.1186/s13099-020-00367-4 32572338 PMC7303439

[55] Derr HT, James MA, Kuny CV, et al Aerosolized hydrogen peroxide decontamination of N95 respirators, with fit-testing and virologic confirmation of suitability for re-use during the COVID-19 pandemic. *medRxiv* 2020 10.1101/2020.04.17.20068577 PMC9599425 36040048

[56] Widmer AF, Richner G. Proposal for a EN 149 acceptable reprocessing method for FFP2 respirators in times of severe shortage. *Antimicrob Resist Infect Control* 2020; 9: 88 published online Jun 17. 10.1186/s13756-020-00744-3 32552867 PMC7298450

[57] Ma QX, Shan H, Zhang CM, Zhang HL, Li GM, Yang RM, et al Decontamination of face masks with steam for mask reuse in fighting the pandemic COVID-19: Experimental supports. *J Med Virol* 2020; published online Apr 22. 10.1002/jmv.25921 32320083 PMC7264583

