# Supplementary material for: Exploring options for reprocessing of N95 Filtering Facepiece Respirators (N95-FFRs) amidst COVID-19 pandemic: A systematic review
Source: PLoS One. 2020 Nov 20;15(11):e0242474. doi: 10.1371/journal.pone.0242474 (PMC7678983; doi:10.1371/journal.pone.0242474)
Supplement: S2 Table — (DOCX) [file pone.0242474.s002.docx]

**S2 Table:** Search Strategy

| **Search engine** | **Search strategies** |
| --- | --- |
| PUBMED | (device, respiratory protective [MeSH Terms]) AND (decontamination [MeSH Terms]), (device, respiratory protective [MeSH Terms]) AND (reuse) [All Fields],  (device, respiratory protective [MeSH Terms]) AND (reusability) [All Fields],  ((device, respiratory protective[MeSH Terms]) ) AND (Reprocessing) [All Fields], ((Reuse) [All Fields] AND (Mask) [All Fields]) NOT (Laryngeal mask) [All Fields], (Reuse) [All Fields] AND (Facemask) [All Fields], (reuse) [All Fields] AND (N95) [All Fields], (reuse) [All Fields] AND (N95 Respirator) [All Fields], (Reuse) [All Fields] AND (Filtering Facepiece Respirator) [All Fields], (Reuse) [All Fields] AND (FFR) [All Fields]  ((Reprocessing) [All Fields] AND (Mask) [All Fields]) NOT (Laryngeal Mask) [All Fields], (Reprocessing) [All Fields] AND (Facemask) [All Fields], (reprocessing) [All Fields] AND (respirator) [All Fields], (Reprocessing) [All Fields] AND (Filtering Facepiece Respirator) [All Fields], (Reprocessing) [All Fields] AND (N95 Respirator) [All Fields], (Reprocessing) [All Fields] AND (N95) [All Fields], (Reprocessing) [All Fields] AND(FFR) [All Fields]  ((decontamination[MeSH Terms]) AND (mask) [All Fields]) NOT (Laryngeal Mask) [All Fields], ((decontamination[MeSH Terms]) AND (respirator) [All Fields], ((decontamination[MeSH Terms]) AND (Filtering Facepiece Respirator) [All Fields], ((decontamination[MeSH Terms]) AND (N95 Respirator) [All Fields], ((decontamination[MeSH Terms]) AND (N95) [All Fields], ((decontamination[MeSH Terms]) AND (FFR) [All Fields]  ((reusability) [All Fields] AND (mask) [All Fields]) NOT (laryngeal mask) [All Fields], (Reusability) [All Fields]AND (Facemask) [All Fields], (reusability) [All Fields] AND (N95) [All Fields], (reusability) [All Fields] AND (N95 Respirator) [All Fields], (Reusability) [All Fields] AND (Filtering Facepiece Respirator) [All Fields], (Reusability) [All Fields] AND (FFR) [All Fields] |
| Google Scholar and Crossref (using Publish or Perish software)/Ovid/  ScienceDirect/  OpenGrey | Decontamination AND FFR  Decontamination AND Respirator  Decontamination AND N95  Reprocessing AND FFR  Reprocessing AND Respirator  Reprocessing AND N95 |
