# Supplementary material for: Exploring options for reprocessing of N95 Filtering Facepiece Respirators (N95-FFRs) amidst COVID-19 pandemic: A systematic review
Source: PLoS One. 2020 Nov 20;15(11):e0242474. doi: 10.1371/journal.pone.0242474 (PMC7678983; doi:10.1371/journal.pone.0242474)
Supplement: S3 Table — (DOCX) [file pone.0242474.s003.docx]

**S3 Table:** Results of Quality Assessment & Risk Bias of Included Studies (After Inter-Author Agreement)

| **Authors** | **Objectives** | **Methodology** | | | | | | **Outcome Measures** | | **Limitations** | **Score** | **Quality** |
| --- | --- | --- | --- | --- | --- | --- | --- | --- | --- | --- | --- | --- |
|  | **Objective/**  **Hypothesis is explicitly stated in the study** | **Are methodological details given, as appropriate, for the decontamination technique such as dose of irradiation, duration of exposure, concentration of the chemical disinfectants, temperature etc.** | **Are classes of respirators explicitly described** | **Sampling methodology clearly defined as per defined objective** | **Sample size calculation described** | **Statistical analysis is done to reject or accept hypothesis** | **Is control group used to compare results** | **Primary outcome measures clearly defined** | **Was the primary outcome measured objectively rather than subjectively** | **Are limitations of the study discussed in relevant section** | **Combined scores based on assessment by both the authors** | **Quality of studies based on scoring** |
| Lindsley et al 2015^8^ | Y | Y | Y | Y | N | Y | Y | Y | Y | Y | 9 | Good |
| Bergman et al 2010^14^ | Y | Y | Y | Y | N | Y | Y | Y | Y | N | 9 | Good |
| Lore et al 2011^15^ | Y | Y | Y | Y | N | Y | Y | Y | Y | Y | 9 | Good |
| Viscusi et al 2009^16^ | Y | Y | Y | Y | N | Y | Y | Y | Y | Y | 9 | Good |
| Fisher et al 2011^17^ | Y | Y | Y | Y | N | Y | Y | Y | Y | Y | 9 | Good |
| Heimbuch et al 2014^18^ | Y | Y | Y | Y | N | Y | N | Y | Y | Y | 7 | Moderate |
| Mills BS et al 2018^19^ | Y | Y | Y | Y | N | Y | Y | Y | Y | Y | 9 | Good |
| Heimbuch et al 2011^20^ | Y | Y | Y | Y | N | Y | Y | Y | Y | Y | 9 | Good |
| Viscusi et al 2011^21^ | Y | Y | Y | Y | Y | Y | Y | Y | Y | Y | 10 | Good |
| Viscusi et al 2007^22^ | Y | Y | Y | Y | N | Y | Y | Y | Y | N | 8 | Good |
| Bergman et al 2011^23^ | Y | Y | Y | Y | N | Y | Y | Y | Y | N | 9 | Good |
| Fisher et al 2010^24^ | Y | Y | Y | Y | N | Y | Y | Y | Y | Y | 9 | Good |
| Vo et al 2009^25^ | Y | Y | Y | Y | N | Y | Y | Y | Y | Y | 9 | Good |
| Fisher et al 2009^26^ | Y | Y | Y | Y | N | Y | Y | Y | Y | Y | 9 | Good |
| Salter et al 2010^27^ | Y | Y | Y | Y | N | N | N | Y | Y | Y | 7 | Moderate |
| Lin et al 2017^28^ | Y | Y | Y | Y | N | Y | Y | Y | Y | Y | 9 | Good |
| Lin et al 2018^29^ | Y | Y | Y | Y | N | Y | Y | Y | Y | N | 7 | Moderate |

**Legend: Scoring Pattern:** 1 Marks for each Y, 0 Mark for Each N. **Grading of studies** – Score (8-10): Good quality, Score (6-7): Moderate quality, Score (≤5): Poor quality
