# Supplementary material for: Exploring options for reprocessing of N95 Filtering Facepiece Respirators (N95-FFRs) amidst COVID-19 pandemic: A systematic review
Source: PLoS One. 2020 Nov 20;15(11):e0242474. doi: 10.1371/journal.pone.0242474 (PMC7678983; doi:10.1371/journal.pone.0242474)
Supplement: S4 Table — (DOCX) [file pone.0242474.s004.docx]

**S4 Table:** Summary of various reprocessing parameters evaluated for specific FFR models

(where disclosed in included studies) by various reprocessing methods

| **RESPIRATOR**  **MODEL** | **RESPIRATOR TYPE** | **PHYSICAL METHODS** | | | | | **LIQUID METHODS** | |
| --- | --- | --- | --- | --- | --- | --- | --- | --- |
|  |  | **UVGI** | **MGS** | **MHI** | **MHA** | **TERC** | **BLEACH** | **ALCOHOL** |
| **3M 1860** | Surgical | P ^21^  O ^21^  D ^21^  F ^21^  E ^8,15^  M ^15,19^ | P ^21,23^  O ^21^  D ^21^  F ^21,23^ | P ^21,23^  O ^21^  D ^21^  F ^21,23^ |  |  |  |  |
| **3M 1870** | Surgical | P ^21,23^  O ^21^  D ^21^  F ^21,23^  E ^15^  M ^15,19^ | P ^21,23^  O ^21^  D ^21^  F ^21,23^  E ^17^  M ^17^ | P ^21,23^  O ^21^  D ^21^  F ^21,23^ |  |  |  |  |
| **3M 8000** | Particulate | P ^21,23^  O ^21^  D ^21^  F ^21,23^ | P ^21,23^  O ^21^  D ^21^  F ^21,23^ | P ^21,23^  O ^21^  D ^21^  F ^21,23^ |  |  |  |  |
| **3M 8210** | Particulate | P ^21,23^  O ^21^  D ^21^  F ^21,23^  E ^24^  M ^24,29^ | P ^21,23^  O ^21^  D ^21^  F ^21,23^ | P ^21,23^  O ^21^  D ^21^  F ^21,23^ | E ^28^  M ^29^ | E ^28^  M ^29^ | E ^28^  M ^29^ | E ^28^  M ^29^ |
| **3M 9210** | Surgical | E ^8^ |  |  |  |  |  |  |
| **3M Vflex 1805** | Surgical | M ^19^ |  |  |  |  |  |  |
| **AP 695** | Surgical | M ^19^ |  |  |  |  |  |  |
| **Cardinal N95-ML** | Surgical | E ^24^  M ^24^ |  |  |  |  |  |  |
| **Gerson 1730** | Surgical | E ^8^  M ^19^ |  |  |  |  |  |  |
| **KC 46727** | Surgical | E ^8^ |  |  |  |  |  |  |
| **KC PFR95-174** | Surgical | E ^24^  M ^24^ |  |  |  |  |  |  |
| **KC PFRN95-270** | Surgical | P ^21,23^  O ^21^  D ^21^  F ^21,23^ | P ^21,23^  O ^21^  D ^21^  F ^21,23^ | P ^21,23^  O ^21^  D ^21^  F ^21,23^ |  |  |  |  |
| **Moldex 1512** | Surgical | M ^19^ |  |  |  |  |  |  |
| **Moldex 1712** | Surgical | M ^19^ |  |  |  |  |  |  |
| **Moldex 2200** | Particulate | P ^21^  O ^21^  D ^21^  F ^21^ | P ^21^  O ^21^  D ^21^  F ^21^  E ^17^  M ^17^ | P ^21^  O ^21^  D ^21^  F ^21^ |  |  |  |  |
| **Moldex EZ-22** | Particulate | M ^19^ |  |  |  |  |  |  |
| **PA RP88020** | Surgical | M ^19^ |  |  |  |  |  |  |
| **Precept 65-3395** | Surgical | M ^19^ |  |  |  |  |  |  |
| **Sperian HC-NB095** | Surgical | M ^19^ |  |  |  |  |  |  |
| **Sperian HC-NB295F** | Surgical | M ^19^ |  |  |  |  |  |  |
| **Wilson SAF-T-FIT Plus** | Surgical | E ^24^  M ^24,25^ |  |  |  |  | M ^25^ |  |
| **US Safety AD2N95A** | Surgical | M ^19^ |  |  |  |  |  |  |
| **US Safety AD4N95** | Surgical | M ^19^ |  |  |  |  |  |  |

**ABBREVIATIONS:** **P**- Physical, **O**-Odour, **D**-Donning Ease & Wear Comfort, **F**-Respirator Fit, **E**: Filter Efficiency, **M**-Microbicidal Efficacy

**RESPIRATOR MANUFACTURERS:**

**3M**: 3M Company, Minneapolis MN

**AP**: Alpha Protech, Markham, Canada.

**Cardinal:** Cardinal Health, Inc, Dublin.

**Gerson:** Lois M Gerson Co, Inc, Middleboro, MA Inc,

**KC:** Kimberly Clark, Halyard Health Inc., Alpharaetta, GA.

**Moldex:** Moldex, Culver city, CA.

**PA**: Prestige Ameritech, North Richland Hills, TX.

**Precept**: Precept Medical products, Inc, Arden, NC.

**Sperian:** Honeywell Safety Products USA, Smithfield, RI.

**Wilson:** Wilson, Santa Ana, CA.

**US Safety:** Dentech Safety Specialists, Lenexa, KS.
