# Supplementary material for: Exploring options for reprocessing of N95 Filtering Facepiece Respirators (N95-FFRs) amidst COVID-19 pandemic: A systematic review
Source: PLoS One. 2020 Nov 20;15(11):e0242474. doi: 10.1371/journal.pone.0242474 (PMC7678983; doi:10.1371/journal.pone.0242474)
Supplement: Attachment — Submitted filename: Response to Reviewers.docx [file pone.0242474.s005.docx]

To

The Editor

PLOSONE

Sir

At the outset we would like to thank the Reviewer(s) for critically reviewing our manuscript in a fair and frank manner. Here is our point by point rebuttal to the suggestions/ concerns raised by the Reviewer:

**Comment 1: Section: Introduction**

The comments “full set of personal protective equipment (PPE) including gowns, gloves, facemasks, face-shields or goggles and respirators for their protection during patient care” and “HCWs need to be protected against bioaerosols at all costs, which at minimum, is offered by use of N95 Filtering Facepiece Respirator (N95-FFR)” are misleading. In fact, citation (4) by the authors states that N95s are only needed during aerosol generating procedures. The authors should clarify this in the introduction.

**Author’s Reply:** The authors have suitably modified the sentence as suggested by the reviewers in the Introduction section (Page 4, Paragraph 1).

**Comment 2: Section: Introduction**

removes > 95% particles of around 300 nm” – there is an official definition of what an N95 filters, the authors should use that

**Author’s Reply:** The authors have modified the sentence and provided the reference of the official definition (Reference 7). In previously submitted manuscript, reference 5 & 7 were duplicated, now the new reference is given in place of Reference 7 (Page 4, Paragraph 1).

**Comment 3: Section: Discussion**

The statement “No independent study prior to 2020 has evaluated microbicidal efficacy of H2O2 on FFRs” is not correct, see Fischer et. al 2020, among others.

**Author’s Reply:** Fischer et al (2020) have referenced Batelle report which evaluated microbicidal efficacy of Hydrogen peroxide for decontamination of N95-FFRs. It was a third party “commercial” evaluation of Clarus C Hydrogen Peroxide vapor generator done by Batelle Institute on behalf of FDA. This wasn’t an independent study published in scientific domain. Hence, we are of the opinion that the statement is correct. Furthermore, we have referenced findings of Batelle report in the Discussion section of the manuscript, where deemed suitable (Page 42, Paragraph 1).

**Comment 4: Section: Discussion**
The discussion is far too long, essentially putting the tables into text. As such:

i. Paragraph 1 of the discussion is off topic and tangential opinion, it should be removed

**Author’s Reply:** In our opinion, this paragraph is important as it highlights the chronology of the scientific progress made in finding a suitable reprocessing method for enabling reuse of FFRs. We humbly opine that had such a method been found in the previous decade, the crises of FFRs wouldn’t have been of this humongous proportions during COVID-19 pandemic. In late to 2000s to late 2010s, there was scientific thrust in finding such a reprocessing method which got derailed in the mid to late 2010s contributing partly to the current crises of shortage of FFRs.

ii. The paragraph that starts “A typical N95-FFR consists of facepiece…” was not material analysed by the literature search and should be removed.

**Author’s Reply:** The paragraph has been removed as suggested by the reviewers (Omitted).

iii. The statement “N95-FFRs are difficult to decontaminate owing to the porous nature of the main body and electrostatically charged nature of electret media” needs a reference, this is debatable

**Author’s Reply:** The statement & paragraph containing it has been removed in the revised Discussion (Omitted).

iv. The discussion needs to be reduced to key points and synthesis, with a cohesive analysis of the literature. At least two pages could be removed.

**Author’s Reply:** Discussion has been extensively modified as suggested by the reviewers.

**Comment 5: Section: Conclusion**

The conclusion doesn’t provide any conclusion regarding the review, but comments on the need for the review and then, again, summarizes the general concepts. There is no analysis here. There is also a dichotomy here, with one sentence stating a need for solution for low income countries (which I agree with), to the very next sentence stating an urgent need to study UV and HPV (not low income country solutions, by the authors own admission).

**Author’s Reply:** The conclusions are now modified, hopefully to the reviewer’s satisfaction. The first 2 paragraphs summarize the findings of the study whereas the last paragraph gives general concepts and way for the future (Pages 45-46).

**Comment 6: Section:**
My biggest concern here is that this really isn’t a systematic review. It attempts to be, and provides the needed supplementary info (i.e. PRISMA diagram) but doesn’t provide any significant analysis. This is really a narrative review with an extensive literature search.

Author’s Reply: We wholeheartedly accept the criticism of the reviewer. However, we will like to counter on the following points:

1. Systematic reviews address a specific research question using explicit methodology of collecting, selecting and appraising studies and synthesizing the results qualitatively or quantitatively, as appropriate.
2. Our review appears as a narrative review because it addresses a broad research question and the outcome of the included studies is discussed more in “Qualitative terms” rather than quantitatively.
3. We are of the opinion that the included studies varied extensively in terms of heterogeneity hence discussion in quantitative terms will not do justice to the essence of the article. Hence, qualitative results of included studies were taken into account in discussion and Quantitative details are provided in the Tables.
4. Overall the studies have not shown conflicting results between themselves for a particular reprocessing method despite being extremely heterogeneous. Hence the findings appear as General concepts, when in fact they are qualitative synthesis of the published literature on the topic. Wherever, conflicting results for a particular reprocessing method have been observed amongst studies, we have made an attempt to discuss.
5. Hence, as suggested by the reviewer, we have revised the Discussion & Conclusion sections extensively focusing now more on the qualitative findings pertaining to the review, hopefully to the reviewer’s satisfaction.

**Minor**

**Comment 7:**
The virus name is actually “SARS-CoV-2” not “SARS-COV-2”

**Author’s Reply:** This has been corrected in all the relevant places

**Comment 8:**
Figure 2 legend “plotted against the reprocessing method” – not sure what the authors mean, it is a table, nothing is “plotted”

**Author’s Reply:** The legend has been suitably modified

Hopefully we have made the necessary modifications/corrections as suggested by the Reviewer(s). We hope for a favourable response from your end.

Thank You

Dr. Ayush Gupta

Assistant Professor

Department of Microbiology

AIIMS Bhopal
