# Supplementary material for: Exploring options for reprocessing of N95 Filtering Facepiece Respirators (N95-FFRs) amidst COVID-19 pandemic: A systematic review
Source: PLoS One. 2020 Nov 20;15(11):e0242474. doi: 10.1371/journal.pone.0242474 (PMC7678983; doi:10.1371/journal.pone.0242474)
Supplement: Attachment — Submitted filename: Response to Reviewers.docx [file pone.0242474.s006.docx]

To

The Editor

PLOSONE

Sir

At the outset we would like to thank the Reviewer(s) for critically reviewing our manuscript. Here is our point by point rebuttal to the suggestions/ concerns raised by the Reviewer:

**COMMENT 1: SECTION: TITLE**

**Please check the term “systematic” in the title, and consider whether it may be changed to “Narrative review.**

**Author’s Reply:** In our humble opinion, a “Systematic review” differs from a “Narrative review” in the following points:

a. The research question is well defined.
b. There is clearly defined criteria for selection for articles published in literature
c. Methods of extraction of and synthesis of data are explicit
d. The critical appraisal of the quality of included studies is explicitly done

In summary, systematic reviews differ from narrative reviews in their “Methodology” rather than the “Results”. We are of the opinion that our review ticks all the boxes in this regard. Furthermore the protocol of the article is also registered with the International prospective register of systematic reviews (PROSPERO) beforehand.

We have already explained that the results of our article are synthesized and discussed more in the qualitative terms rather than quantitative terms due to the heterogeneity in the methodologies of various articles who have researched this topic. Hence, we humbly request that we should persist with the word “Systematic” in the title.

**COMMENT 2: SECTION: DISCUSSION**

**The authors must have the wrong Fischer et al. (2020)- I am referring to 1. Fischer R.J., (2020) Effectiveness of N95 Respirator Decontamination and Reuse against SARS-CoV-2 Virus. Emerging Infect Dis 10.3201/eid2609.201524. This article doesn’t reference any other article , but does the experiments themselves. This is but one example. It is actually very important to the manuscript, as this is now the most common method for commercial decontamination.**

**Author’s Reply:** We had cited the same study which the reviewer is referring to but it was a preprint version which we cited (Reference 43). Our statement in previous rebuttal *“Fischer et al (2020) have referenced Batelle report which evaluated microbicidal efficacy of Hydrogen peroxide for decontamination of N95-FFRs”*was based on this pre-print version but their comment about Batelle report are now removed from the final print when this article is published as “Letter” in Emerging Infectious Disease, which the reviewer is referring to, hence the confusion. Thus, we have now modified the reference of this article (No. 43) in our manuscript to final print version from the originally given pre-print version.

In the first cycle of peer review, the reviewer had commented “*The statement- No independent study prior to 2020 has evaluated microbicidal efficacy of H_2_O_2_ on FFRs - is not correct, see Fischer et al 2020, among others*.” We modified it in our article as “*Prior to 2020, no study, in published literature, had evaluated microbicidal efficacy of H_2_O_2_ on FFRs, but recently, Fisher et al[43] found it effective in removing SARS-CoV-2 from N95-FFRs”.* Our statement in the manuscript is correct as probably the phrase “Prior to 2020” is ignored. This study by Fischer et al is published online in June 2020 (preprint version) & final version in September 2020. They evaluated the efficacy of VHP (Vaporized Hydrogen Peroxide) on N95 respirators contaminated with SARS-CoV-2 virus, a virus which was non-existent prior to 2020, so how can our statement be deemed as incorrect as we are specifically talking about research done Prior to 2020.

Furthermore, it is said that such inaccuracies are an example. We humbly request the reviewer to provide specific statements which are inaccurate and require correction.

**Comment 3: Section: Discussion**
The discussion is still too long and re-states the results. I encourage the authors to think of how to discuss the results, not re-state them.

**Author’s Reply:** We humbly request the reviewer’s criticism in terms of 2 things: length of discussion and re-stating the results in discussion, but kindly consider this:

a. The discussion appears lengthy because there are multiple methods which have been evaluated in the published literature for reprocessing of FFRs. We believe that all these methods needs to be discussed individually in terms of their variables, pros, and cons so that their suitability in reprocessing of FFRs in a particular setting can be assessed.
b. Regarding, the re-stating of results in discussion section, we believe that some form of re-stating of the result is required to initiate discussion of a particular issue.

Hence, taking the criticism in our stride, we have attempted to reduce the length of the discussion, hopefully to the reviewer’s satisfaction. Overall word count of our manuscript text (without abstract, tables/ figures and their legends, references) is 4633, which in our opinion is usual for such articles.

Dr. Ayush Gupta

Assistant Professor

Department of Microbiology

AIIMS Bhopal
